# Strengthening geriatric oral health care through interprofessional education: policy and practice implications

**DOI:** 10.3389/fdmed.2026.1784205

**Published:** 2026-04-13

**Authors:** Haziel Diana Jenifer, Vinita Boloor, Wasim Belim

**Affiliations:** Department of Periodontology, Yenepoya Dental College, Yenepoya (Deemed to be University), Deralakatte, Mangalore, Karnataka, India

**Keywords:** aging and oral health, curriculum reform, geriatric dentistry, interprofessional education, policy review

## Abstract

**Background:**

The global population is aging rapidly, and India is entering a phase of rapid demographic transition, similar to already super-aged societies like Japan and several European nations. As a result, older adults are increasingly retaining their natural dentition while living with multimorbidity, polypharmacy, cognitive decline, and functional impairment. However, dental education systems remain largely procedure-centric and disconnected from medicine and allied health. This misalignment has significant implications for patient safety, workforce readiness, and the sustainability of the health system.

**Objective:**

This Policy & Practice Review critically examines existing policies, accreditation standards, and educational practices governing geriatric oral health education, with a specific focus on India and global comparative perspectives. It evaluates interprofessional education (IPE) as a policy tool for curricular reform and assesses its implications for regulators, academic institutions, and health systems.

**Methods:**

A structured narrative policy review was conducted utilizing peer-reviewed literature from PubMed and Scopus, alongside official global and national repositories. Evidence was synthesized from World Health Organization (WHO) frameworks, international accreditation standards (e.g., CODA, ADEE, IPEC), and Indian national policies (NPHCE, Ayushman Bharat) from 2010 to 2025. Documents were evaluated based on their relevance to geriatric competencies, interprofessional education (IPE) models, and health system outcomes in India and selected comparator nations (Japan and Brazil)

**Findings:**

Across regions, a persistent “geriatric gap” in dental curricula is evident, characterized by insufficient exposure to frail older adults, inconsistent competency definitions, and limited integration with primary care and long-term care systems. Countries that have incorporated oral health within healthy aging and long-term care policies demonstrate stronger alignment between education and practice. Evidence suggests that IPE-based geriatric curricula enhance learner attitudes, diagnostic reasoning, and care coordination, leading to improved patient outcomes.

**Actionable recommendations:**

To address these challenges, it is crucial to mandate interprofessional geriatric competencies within accreditation standards, integrate oral health into national healthy aging policies (including India's National Programme for Health Care of the Elderly), and align reimbursement with preventive, team-based care.

**Conclusion:**

Geriatric oral health education must be prioritized as a policy and practice imperative. Coordinated reforms across education, regulation, and financing are essential to build an age-ready oral health workforce.

## GRAPHICAL ABSTRACT

**Figure:**
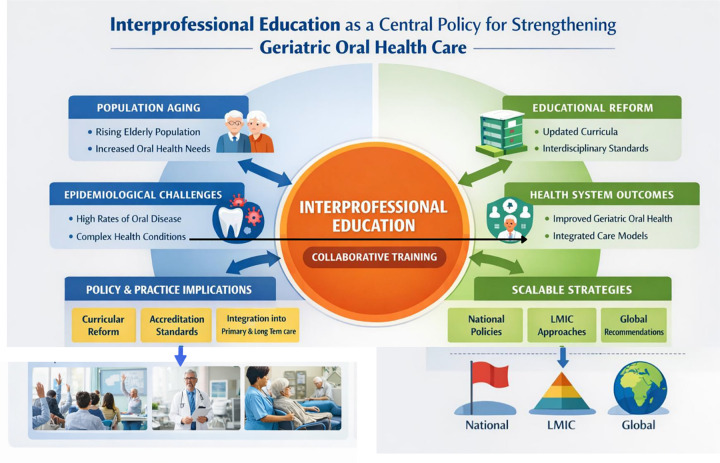
Infographics showing the interprofessional education as a central policy mechanism linking population aging and epidemiological challenges with educational reform and health-system outcomes. The infographic highlights key policy and practice implications, including curricular reform, accreditation standards, integration of oral health into primary and long-term care, and differentiated applicability at national, LMIC, and global levels.

## 1 Introduction

The population ageing is considered one of the major challenges in the field of global health in the twenty-first century. According to the World Health Organization (WHO), the number of individuals aged 60 years and older is expected to grow from 1 billion in 2019 to over 2.1 billion in 2050, with close to 80% of this increase expected to occur in low- and middle-income countries (LMICs) (1, 2). The aging of the population is not only a challenge in terms of its magnitude but also in terms of its rapidity, which is expected to compress transitions that took a century in high-income countries into a few decades (1, 3). As a result, health systems, professional education structures, and policy frameworks are under increasing pressure to adapt to the complex needs of aging populations.

The scale and complexity of this shift can be understood through the example of India. According to United Nations projections, the population aged ≥60 years in India is expected to increase from around 149 million in 2022 to over 300 million in 2050, which would be close to one-fifth of the total population (3, 4). Unlike other nations that experienced demographic aging after realizing overall socioeconomic development, India faces the challenge of aging populations along with the challenges of health inequities, lack of integrated service delivery, and significant out-of-pocket expenditure. These realities have important implications for how geriatric health—including oral health—is conceptualized, prioritized, and delivered within the health system (4, 5).

Oral health is a paradoxical yet highly important area of concern for healthy aging. On one hand, the development of preventive dentistry, fluoridation, and restorative dentistry has allowed many older individuals to maintain their natural dentition well into their advanced years (6, 7). However, this success has been achieved at the expense of an increasingly cumulative burden of chronic diseases of the oral cavity, including periodontal disease, root caries, tooth wear, peri-implant disease, and oral mucosal disease (6, 8). These conditions rarely occur in isolation. They have a bidirectional relationship with systemic diseases such as diabetes mellitus, cardiovascular disease, chronic respiratory disease, and neurocognitive disorders, which are common in older individuals (7, 8).

From a public health perspective, the consequences of poor oral health in the elderly have implications that go well beyond the dental office. Epidemiological studies have shown that poor oral hygiene is a risk factor for aspiration pneumonia in the elderly, tooth loss and malnutrition, and untreated oral pain and functional impairment (9, 10). These complications are associated with hospitalizations, increased institutionalization, and a reduced quality of life, thus adding to the economic burden on families and the healthcare system (10, 11). In rapidly aging societies, the failure to address oral health issues in geriatric care is a lost opportunity for cost containment (9, 11).

Despite this evidence, oral health remains marginal within most national aging and noncommunicable disease policies. The WHO has repeatedly stressed that oral health is an essential component of general health, well-being, and quality of life throughout the life course (1, 12). Recent WHO resolutions have emphasized the integration of oral health into universal health coverage and primary health care, recognizing its relevance to healthy aging (12, 13). However, the implementation of these global policy goals into national programs and professional education has been patchy, especially in LMICs (13, 14).

The dental education infrastructure reflects this policy marginalization. Traditionally, dental education has been structured around discipline-specific technical skills with minimal focus on managing complex chronic diseases, functional evaluation, and teamwork. Geriatric dentistry, when it exists, tends to be dispersed across topics such as prosthodontics, periodontology, oral medicine, or public health dentistry, lacking a well-integrated competency framework that addresses issues of frailty, cognitive impairment, ethical consent, or teamwork (6, 15). Thus, graduates may be skilled in procedures but not adequately equipped to deal with the realities of aging and multimorbidity and dependency (15, 16).

In India, these educational gaps are particularly consequential. The Dental Council of India (DCI) prescribes a uniform curriculum for undergraduate and postgraduate dental education, but geriatric dentistry is not recognized as a distinct competency domain or specialty (17, 18). Exposure to older adults is largely opportunistic and clinic-based, often limited to relatively healthy, ambulatory patients who do not reflect the broader geriatric population (17, 18). Moreover, formal interaction with medical, nursing, or allied health trainees during dental education remains minimal, reinforcing professional silos at precisely the stage when collaborative identities are formed (19, 20).

National health policies further extend these realities of health education. Programme for Health Care of the Elderly (NPHCE) was established to provide comprehensive geriatric services through primary, secondary, and tertiary care facilities (21, 22). However, oral health is conspicuously absent from NPHCE service delivery models and workforce training guidelines (21, 22). Similarly, while Ayushman Bharat and Health and Wellness Centres emphasize comprehensive primary care across the life course, oral health integration remains limited and inconsistently implemented (23, 24). This policy silence signals to educational institutions that geriatric oral health is peripheral rather than foundational to healthy aging.

International experience suggests that such policy and educational disconnects are neither inevitable nor benign. Countries that have explicitly integrated oral health into aging and long-term care policies demonstrate stronger alignment between education, service delivery, and population needs. Japan, for example, has embedded oral function management within its Long-Term Care Insurance system, with reimbursement structures that support interprofessional collaboration and home-based dental care (25, 26). These policy signals have, in turn, shaped dental education toward functional, team-based geriatric practice. In contrast, systems that rely primarily on specialty recognition without broader integration risk limiting access and scalability, particularly in rural and underserved areas (27, 28).

In order to address the challenge of aging, it is essential to establish a clear definition of Interprofessional Education (IPE) as a driver for health system reform. According to the World Health Organization (WHO, 2010) (29, 30) and the Interprofessional Education Collaborative (IPEC, 2016), IPE occurs when “students from two or more professions learn about, from, and with each other to enable effective collaboration and improve health outcomes”. It is critical to distinguish IPE from multiprofessional learning, where students from different disciplines may share a physical space or a lecture but continue to learn in “silos” without meaningful interaction or integrated problem-solving (29, 31).

In the field of geriatric oral health, interprofessional education (IPE) can be divided into two different educational models:

1. Classroom-based IPE (Didactic): This model includes theoretical learning together, such as case discussions or seminars where dental, medical, and nursing students work together to develop care plans for complex geriatric patients with multimorbidity and polypharmacy. While very effective for knowledge transfer, these models are often introductory in nature (20).
2. Clinical IPE (Integrated Practice): This model includes rotations in high-intensity clinical environments such as geriatric hospitals, long-term care facilities, or home health agencies where students engage in collaborative clinical decision-making (20).

Interprofessional education (IPE) has emerged globally as a critical policy instrument to address these challenges. Defined as learning “with, from, and about” other health professions, IPE is strongly endorsed by the WHO as a means to strengthen health systems and improve collaborative practice (29, 30). In geriatric care, where oral health intersects with medical management, nutrition, mobility, and social support, interprofessional competence is not optional but essential. Evidence from dental and medical education demonstrates that IPE improves learner attitudes toward older adults, enhances understanding of oral–systemic interactions, and strengthens communication and teamwork skills relevant to patient safety (31, 32).

First and foremost, it is important to recognize that IPE is not just an educational tool but rather a transformation lever. IPE can integrate into criteria of accreditation and licensure on a national level to redesign professional identity by incorporating interprofessional competencies that embed collaboration into professional practices. Otherwise, the development of IPE programs can be left isolated without mandates.

Evidence suggests that the Clinical IPE models have the most significant impact on geriatric care. More specifically, the care models that include oral health professionals in geriatric teams have shown significant reductions in the development of aspiration pneumonia and improvements in nutritional status in vulnerable older adults (10, 30). Through the progression from classroom exposure to integrated clinical practice, the healthcare system can progress from disintegrated care to a cohesive, age-friendly approach that supports patient safety and sustainability (32).

This Policy & Practice Review reframes geriatric oral health education as a matter of governance rather than curriculum alone. It makes an attempt to point that the workforce preparedness are predictable outcomes of policy choices that marginalize oral health within aging agendas and permit ambiguity within accreditation standards (1, 35). By adopting an India-plus-global comparative lens, the review examines how different policy environments shape educational priorities and practice realities, drawing lessons from both high-income and middle-income contexts (2, 35).

There are three major objectives of the review. The first aim involves systematically identifying existing international and national policies, accreditations, and guidelines concerning the education of geriatric oral health. The second aim focuses on the implications of the aforementioned for clinical practice and the performance of the healthcare system; the target involves inter-professional practice and long-term care. The third aim includes formulating recommendations based on policy.

By integrating elderly oral health education with overall objectives related to healthy aging, universal health insurance, and workforce reform, the present literature review seeks to arrive at a more holistic and balanced understanding related to elderly oral health issues. In rapidly aging societies such as India, the stakes of inaction are high. Aligning dental education with demographic reality is not only an educational imperative but a public policy necessity.

## 2 Methodology

The review concentrated on documents and studies that were published between 2010 and 2025 to ensure that the modern development of aging policies and the development of IPE as a global healthcare standard was covered. The review was global in nature, but it mainly concentrated on the Indian healthcare system as the main case study. This was supplemented by the policy pathways of Japan and Brazil.

### 2.1 Inclusion and exclusion criteria

Documents were selected based on the following criteria:

**Inclusion Criteria:**

- National health policies and program operation guides (for example, NPHCE, Ayushman Bharat)
- Accreditation standards that explicitly reference geriatric or special-needs competencies
- Systematic reviews that explore how oral health influences broader outcomes (such as aspiration pneumonia and hospitalizations)
- IPE frameworks endorsed by international bodies like IPEC.

**Exclusion Criteria:**

- Technical clinical studies that focus only on surgical or prosthetic techniques without any educational or policy context
- Sources not available in English
- Editorial opinions that cannot be verified through institutional or peer-reviewed support

The data was synthesized by matching educational competencies with health system outcomes. The review also utilized the 4Ms framework (What Matters, Medications, Mentation, Mobility) as a secondary lens to assess the age-friendliness of current dental curricula and their integration with overall care models.

## 3 Assessment of current policies and guidelines evaluation

### 3.1 Global policy and accreditation landscape

The educational agenda in the field of dentistry operates largely under the umbrella of accreditation standards, professional regulation, and policies influencing the field of healthcare and education at a global level and within each country. However, in the setting of rapid population aging, their influence becomes especially important, as unclear or non-specific standards may leave the workforce inadequately prepared to manage complex geriatric needs (1, 3).

At the global level, the World Health Organization (WHO) has consistently emphasized that oral health is an integral component of healthy aging, universal health coverage, and primary health care (1, 2). The WHO's World Report on Aging and Health positions functional ability, rather than the absence of disease, as the end state of health systems for older adults (1). Oral health directly affects functional ability through its impact on nutrition, communication, social participation, and the preservation of dignity (1, 8). Despite this conceptual integration, oral health remains poorly operationalized in many national aging strategies, particularly in workforce education and competency frameworks (12, 13).

Establishing accreditation requirements is also a key factor in determining the quality of dental education programs in wealthier countries. In the United States and Canada, the Commission on Dental Accreditation (CODA) mandates that graduating dental students demonstrate proficiency in treating patients with special healthcare needs, including older individuals (15). CODA, however, maintains flexibility in its requirements and does not specify minimum clinical experience for frailty or dementia. Additionally, it does not set requirements for interprofessional clinical exercises (6, 15). These adaptable criteria lead to variations across institutions and result in differences in graduates' competency levels. Prior research indicates significant inconsistency in how dental programs teach about older adults, ranging from extensive clinical rotations in long-term care settings to classroom instruction towards the end of their training (15, 16).

The same patchwork is evident in the European regulatory landscape. The Association for Dental Education in Europe (ADEE) has developed a profile for the European graduate dentist that includes skills in caring for older patients and those with special needs (16). In addition, the European College of Gerodontology (ECG) has developed detailed undergraduate curriculum guidelines on aging biology, multimorbidity, cognitive decline, ethical consent, and care in care-home settings (6). However, these guidelines remain advisory and are not mandatory. National accreditation bodies are responsible for implementation, which is often influenced by institutional priorities. Consequently, the actual adoption of these standards varies significantly across Europe. Nordic countries, where social care is more accessible, typically offer a more comprehensive geriatric training program compared to countries where dental education remains exclusively clinic-focused (15).

In low- and middle-income countries (LMICs), formal accreditation standards are also less specific, and enforcement capacity may be weaker. Dental curricula emphasize procedural skills and high-volume clinical requirements given workforce needs and service delivery pressures. When geriatric oral health is included, it typically falls under community dentistry or prosthodontics, with no explicit focus on frailty, dependency, or interprofessional collaboration (18). Such limitations limit the view of dental practice and conflict with the demographic realities of aging populations (22).

From a policy perspective, a significant structural obstacle to reform is that these geriatric competencies are not explicitly mentioned in the accreditation requirements. When accrediting bodies do not mandate them, it is unlikely that there will be regular teaching, testing, or funding of the subject. Consequently, regional disparities emerge due to the voluntary nature of the efforts at the institutional level (6, 13).

### 3.2 Interprofessional education frameworks and policy significance

During the past two decades, interprofessional education (IPE) has emerged as a fundamental driver for health workforce change. Based on the World Health Organization's Framework for Action on Interprofessional Education and Collaborative Practice, interprofessional education is viewed as an enabler to strengthen health systems, better health outcomes for patients, and increase the effectiveness of the workforce (29). Complementing this, the Interprofessional Education Collaborative has provided fundamental guides for implementing interprofessional education that cover the following four primary areas: values/ethics for interprofessional practice, roles and responsibilities, interprofessional communication, and teams and teamwork (31).

In terms of policies, these guidelines are significant because they enable high-level objectives for collaboration to be integrated into specific competencies that can be utilized in accreditation and licensure. In geriatric care, where patients necessitate the collective input of various professionals, these competencies are crucial. Topics related to oral health encompass medication management, nutritional concerns, cognitive function, mobility, and social support—areas that have traditionally been beyond the scope of dental care (29, 31).

The body of evidence supporting Interprofessional Education (IPE) in a geriatric context is growing. Systematic reviews of health profession education have shown that IPE positively impacts learners' attitudes towards teamwork, reinforces their professional identity, and enhances communication skills. In particular, within the realm of dental education, interprofessional curricula in geriatrics have been linked to increased confidence in providing care to medically complex elderly patients, a better understanding of the relationship between the oral system and overall health, and preparation for collaborative practice (20). It's worth noting that these educational outcomes align with policy initiatives aimed at improving patient safety and quality of care (29).

Despite the evidence, dental accreditation still lacks consistent integration of Interprofessional Education (IPE). While IPE is encouraged voluntarily, it is often confined to the classroom when it becomes a mandatory component. This raises concerns about the governance of education, as a structured education system and adequate funding are essential for interprofessional education to progress and make meaningful changes (20, 31).

### 3.3 Indian policy and regulatory framework

In India, the dental education and health landscape is a complex blend of government-regulated policies and fragmented programs. This structure is primarily governed by the Dental Council of India (DCI), which establishes educational programs for both undergraduate (BDS) and postgraduate (MDS) courses. While this standardization aims to provide a consistent framework, it also limits flexibility in adapting to demographic changes, particularly the growing elderly population (17).

Currently, the DCI structure neglects to recognize elderly dental care as a distinct area of practice or competency. Consequently, the content related to elderly dental care is spread across various topics, including prosthodontics, public health dental practice, oral medicine, and periodontics. While these subjects may impart basic knowledge about elderly dental concerns, they fail to present a comprehensive and cohesive strategy for addressing the unique needs of the elderly population. Key domains such as assessing vulnerability, dementia practice, managing polypharmacy, obtaining ethical consent, and fostering collaboration are often covered inadequately or not at all (17, 18).

National health policy instruments shape the focus on aging care. The Indian government's primary initiative, the National Programme for Health Care of the Elderly (NPHCE), aims to provide comprehensive care for the elderly across primary, secondary, and tertiary care facilities. However, oral health care is notably absent from the service areas and performance areas of the NPHCE program. This oversight highlights the potential consequences of neglecting oral health care in aging policies, leading to the marginalization of dental education in the context of aging care (22).

The National Health Policy and the Ayushman Bharat Yojana emphasize the importance of continuous primary healthcare throughout life, including for older individuals, through Health and Wellness Centers (23). However, the integration of oral healthcare in these centers is limited due to manpower shortages and policy ambiguities regarding the role of dentists in providing primary care to older adults (23, 24).

Policy analysis reveals that India's experience demonstrates the challenges of implementing vertically designed disease control strategies in achieving good oral health. Geriatric strategy outreach primarily focuses on managing chronic diseases, leaving the field trapped in a dentistry educational and service box. Bridging this gap requires a policy that integrates dentistry education, geriatric care, and interprofessional teamwork (18, 22).

### 3.4 Comparative international models: Japan and Brazil

Analyzing various international approaches to geriatric oral health through education and practice is enlightening. Examples of non-United States models that may offer valuable insights include those of Japan and Brazil.

Japan, the country that pioneered the super-aging model, employs a system-integrated strategy. This strategy integrates a comprehensive oral health management system with their Long-term Care Insurance (LTCI) program. It involves managing oral functions, screening patients for dysphagia, and assisting in maintaining oral health, all of which are covered under long-term care and have favorable reimbursement terms. This collaboration allows dentists to work alongside health professionals in an interdisciplinary team setup (25). These policy signals strongly influence dental education, encouraging curricula that emphasize functional outcomes, home-based care, and collaboration with medical and caregiving professionals (27).

Research from Japan reveals a positive correlation between long-term care integrated with oral health care, improved nutrition, reduced incidence of aspiration pneumonia, and overall quality of life among the elderly population (25, 26). However, the key factor is the development of personnel skill sets within a supportive policy framework, emphasizing a team-working mentality (27).

In contrast, Brazil has a distinct approach, recognizing Geriatric Dentistry (or “Odontogeriatria”) as a dental specialty. This recognition has led to enhanced academic pursuits and advancements in postgraduate education in this field. However, the challenge lies in providing access to specialized care in countries experiencing “aging epidemics” (28). In countries with large and rapidly aging populations, such models might exacerbate access disparities unless robust generalist training is provided.

### 3.5 Policy synthesis and strategic implications

By integrating data from around the world, across nations, and in comparative studies, it becomes evident that there are a few fundamental concepts that transcend these diverse contexts. Firstly, for gerontological policies to bring about positive change, the inclusion of oral health in these policies is imperative (1, 3). Without this inclusion, educational reforms will fail to be effectively implemented. Secondly, outlining gerontological competencies in accreditation is crucial to ensure the consistent preparation of the workforce (6, 31). Thirdly, adopting approaches that integrate oral health into primary care and long-term care pathways, rather than focusing on specialization, is more equitable (25, 28).

For countries like India, several implications arise from these findings. A more modest strategy is necessary. Incorporating geriatric oral health skills into existing frameworks may be more advantageous than undertaking a massive transformation. By harmonizing dental education with country-level strategies on aging and providing adequate funding, India can effectively address the oral health challenges posed by its aging population.

## 4 Implications for practice and health systems

Geriatric oral health education extends far beyond the confines of classrooms, profoundly influencing patient care, workforce organization, health system performance, and equitable care provision. As the population ages, the delivery of care to older adults increasingly occurs in medically and socially complex settings characterized by multimorbidity, functional decline, cognitive challenges, and heavy caregiver reliance. If dental education fails to adequately prepare dentists for these realities, it directly impacts patient safety, the quality of care, and the sustainability of health systems (1, 6).

### 4.1 Workforce readiness and clinical practice

Preparing the workforce is a fundamental objective of education policy. Dentists are increasingly being tasked with managing older adults who have multiple chronic diseases, extensive medication regimens, and varying degrees of physical and cognitive dependency. Numerous studies consistently reveal that a significant portion of dental graduates feel inadequately prepared to handle medically complex older patients, let alone those in long-term care or receiving home-based care (6, 18). This disparity should not be attributed to individual shortcomings but rather suggests more systemic issues with the design of education and the establishment of regulatory expectations (17).

Geriatric dental decision-making demands skills that diverge from those emphasized in traditional curricula. Treatment planning must strike a delicate balance between disease control and the preservation of function, comfort, and prognosis, while also considering patient preferences. Polypharmacy poses risks of drug interactions and oral health complications such as xerostomia, hemorrhage, and impaired healing (7, 8). Cognitive impairment further complicates consent and necessitates cooperation with caregivers and legal representatives. Without targeted training in these critical areas, dentists may inadvertently resort to overly aggressive treatments or therapeutic nihilism, both of which can exacerbate patient outcomes (16).

Interprofessional approaches to geriatric teaching have played a pivotal role in elevating the oral health of elderly patients within the broader context of their overall health. By collaborating closely with all healthcare professionals, dentists acquire the ability to interpret medical histories, communicate effectively with doctors and pharmacists, and identify situations where dental practice poses a risk to the overall well-being of their patients (29, 31). The interprofessional approach, where dental and other health education converge, has demonstrated that dentists feel more confident in managing challenging patients and are more inclined to participate in primary health teams (32).

### 4.2 Long-term care facilities and institutional practice

Long-term care facilities (LTCFs) represent a significant and underserved segment of geriatric dental health. Global data reveals a substantial number of untreated dental issues, including caries, gum disease, dental pain, and poorly maintained dental appliances (6, 22). Several insurmountable physical barriers hinder dental care access, professional responsibility definitions, and education for nursing home staff regarding dental health (22, 36).

Neglecting dental care in LTCFs has far-reaching consequences. Poor dental hygiene is linked to an increased risk of aspiration pneumonia, a prevalent cause of morbidity and mortality among vulnerable senior citizens (9, 10). Dental-related distress and denture loss can hinder nutrition and contribute to frailty, while poorly fitting dentures can lead to mucosal ulcerations and infections. These factors collectively result in a significant burden on healthcare systems due to increased hospital admissions and stays (11).

The integration of dentists, supported by policies, has proven beneficial. In Japan, dentists are considered integral to long-term care facility support staff within the Long Term Care Insurance system. They receive reimbursement to contribute to the evaluation of oral health status and dysphagia care (25, 27). Aligning dental education with such policies ensures that dental students are well-prepared to manage patients in health facilities and collaborate effectively with support staff like caregivers (26).

In India, the rise of residential elder-care facilities reflects changes in family dynamics and urbanization. However, monitoring oral health services within these facilities remains limited. Therefore, facilitating appropriate training for dentists to work effectively in LTCFs through interprofessional education is crucial for both the workforce and policy environment (18, 22).

### 4.3 Primary care, community health, and preventive integration

The primary care model offers innovative approaches to seamlessly integrate dental care into the comprehensive geriatric care framework. There's a global shift from disease-focused treatment to preventive measures that maintain individuals' functional status and provide patient-centered care throughout their lifespan (1, 12). Dentists can significantly contribute to this movement by conducting dental screenings for nutritional-related dental issues and identifying early signs of vulnerability or cognitive decline (31).

Within a community, oral health problems can serve as early indicators of broader physical health changes. Symptoms such as weight loss, decreased food intake, or alterations in brushing and hygiene practices may signal impending frailty or depression. By training dentists to monitor these indicators and collaborate with healthcare professionals, patients can receive timely interventions and prevent further complications related to their physical condition (8, 23).

The existing pattern of service delivery in Old Age Homes (OAHs) reflects an integrative gap in medical and dental care, which may indicate an integrative gap in the holistic management of health. While medical care in OAHs is well structured, particularly in managing chronic conditions, dental care remains fragmented or elective in nature. A significant number of OAHs provide medical care, including nursing and doctor consultations, without dental care. This may be due to the lack of dental facilities and the absence of oral health indicators in the licensing and accreditation standards of OAHs in the country. The medical staff at OAHs may not have the training or skills to carry out basic oral screenings or to understand the link between oral health and conditions such as malnutrition and xerostomia (5). Providing medical care without dental care may indicate an integrative gap in the holistic management of health. Oral hygiene among residents at OAHs has been identified as one of the major factors that contribute to aspiration pneumonia, resulting in hospital transfers and mortality among residents (10). The Health and Wellness Centers established by the Indian government under the Ayushman Bharat program provide an ideal platform for such integration (23, 24). However, successful implementation hinges on having a well-trained workforce capable of extending their services beyond the confines of traditional dental clinics. A comprehensive overhaul in education, emphasizing community-based inter-professional care for the elderly, is essential to unlock the potential of integrating primary care and oral health (18).

### 4.4 Economic, financing, and system sustainability implications

From a health-system perspective, oral health education for older adults has some straightforward economic implications. When older adults lack good oral health, they tend to use more health services, such as hospital admissions for conditions that could have been prevented, like aspiration pneumonia, malnutrition complications, and chronic disease exacerbations (10, 11, 37). These factors increase costs and strain already limited systems (22).

We should consider the value of investing in interprofessional geriatric education using a value-based approach. Preventing oral health within integrated care models can reduce downstream costs and improve quality of life. Research suggests that maintaining oral function can delay the need for institutional care and reduces dependency, resulting in savings that extend beyond dentistry (11).

The oral healthcare burden for the geriatric population is a heavy one and a major hindrance in healthy aging. Dental care is an expenditure in both the country and the world. Even in countries like India, dental care for the geriatric population is not included in schemes like Ayushman Bharat. This problem is further compounded by the multimorbidity-poverty trap in which the elderly have to choose between life-sustaining drugs and dental care (38). Financial considerations play an important part in the use of healthcare services. High costs result in delayed care and treatment during emergencies. In super-aged countries, financial barriers are alleviated through a combined reimbursement system, as in Japan through their Long-Term Care Insurance scheme. This increases preventive care and decreases long-term care costs through complications of malnutrition and pneumonia (26). While some individuals may be able to access dental care through Medicaid or private insurance, reimbursement rates for geriatric oral health care are typically inadequate to motivate healthcare providers to treat older individuals (38). Such economic consequences of oral health issues will disproportionately impact older individuals of lower socioeconomic status, racial and ethnic minorities, and those living in rural settings, thereby contributing to existing health disparities (39). Additionally, billing mechanisms do not currently support interdisciplinary care models; that is, physicians cannot be reimbursed for oral health screenings, nor can dentists be reimbursed for coordinating with other healthcare providers. In India, where households bear a significant share of health costs out of pocket, incorporating preventive oral health into geriatric care could alleviate financial burdens for older families. Therefore, developing educational competencies alongside financing structures that reward prevention and teamwork is crucial for the long-term sustainability of the system (21, 22).

### 4.5 Implications for academic institutions and faculty capacity

Educational institutions are currently at a critical juncture in translating policy mandates into practical realities in classrooms and clinical settings. Implementing interprofessional education in geriatrics necessitates revamping the curriculum to foster collaboration among various departments and investing in faculty development. Educators must be prepared to deliver education in team-based contexts and manage the teaching and ethical considerations associated with caring for older individuals (20, 31).

A persistent challenge in the field of education is faculty development. Many dental faculty members have been trained in a closed system and may lack direct experience working in interprofessional or geriatric practices. This underscores the need for national and institutional initiatives that prioritize faculty and staff development, fellowships, and teaching (6, 18).

Without alignment and/or policy support, these efforts risk drifting, stalling, or becoming unsustainable. It is crucial to ensure that there is alignment between the requirements for accreditation, the health priorities, and the leadership. Additionally, these reforms should have the potential to improve practice and performance within the health system.

## 5 Policy boxes and practice implications

Policy boxes serve a critical translational function in Policy & Practice Reviews by distilling complex analyses into actionable insights for policymakers, regulators, educators, and practitioners. In the context of geriatric oral health education, they highlight systemic gaps, comparative lessons, ethical considerations, and practice-level consequences, thereby bridging evidence with implementation (1, 13).

### 5.1 Policy box 1: gaps in the structure and systems related to older persons' oral healthcare learning

Although the aging population and the associated health issues are increasingly recognized and the gerodontodontic branch has developed, certain systemic and structural deficiencies persist in the gerodontodontic education of dentists. These deficiencies are not solely learning-related but also reflect issues related to governance and workforce preparation (6, 18).

Ambiguity in regulation is a key concern. In most places, regulations regarding accreditation for care in older adults and/or patients with special health care needs are required. However, these regulations do not specify the specific competencies required for older adult patients. When goals are vague, their achievement becomes inconsistent (6, 15, 16). Competencies that are not measurable cannot be assessed, developed, or given importance.

Educational silos exacerbate the problem. Historically, dental education has proceeded independently of, rather than in collaboration with, the education of medical, nursing, pharmacy, and allied health professionals. This has resulted in a significant issue, as dental graduates lack a comprehensive understanding of the contributions of other professionals involved in gerontological care (29, 31). In an aging population, the silo education approach is incongruous with the reality of clinical care, as successful care delivery relies on a team effort (20).

The second major gap lies in the lack of alignment between service and education. National policies concerning aging and non-communicable diseases often neglect oral health, placing it outside the mainstream of healthy aging policy initiatives (1, 21).

The limited capacity of faculty members further exacerbates this issue. Most dental faculty members are trained in a traditional procedural approach and may lack experience in caring for older patients or teaching interprofessional practices. Without professional development for a well-structured curriculum, it becomes challenging to transfer knowledge from the classroom to practice (6, 18).

Together, these factors create a cycle where a lack of policy discourse reinforces a failure in educational provision, resulting in an underqualified workforce that fails to meet the demands for good oral healthcare among an aging population.

### 5.2 Policy box 2: comparative policy pathways—India, Japan, and Brazil

A glance at the policies reveals how different courses of action impact education within schools and the treatment of older individuals.

In India, the government is committed to supporting geriatrics through initiatives like the National Programme for Health Care of the Elderly (NPHCE) or Ayushman Bharat (21, 23). However, oral health is often overlooked in these strategies. To ensure consistent dental education, a controlled approach is implemented, although it becomes challenging to function effectively with a diverse population (17, 22).

Japan serves as an example of system-wide integration. Oral health is included in the coverage of Long-Term Care Insurance, and Perc/back claims are provided for managing oral functions, assessing swallowing difficulties (dysphagia), and providing dental care at home (25, 26). These system-wide examples, along with other cues, promote teamwork and functional curriculum development. There is evidence that integrated care improves dietary intake and reduces the risk of aspiration pneumonia (26).

Brazil takes a different approach by recognizing specialties with the formal naming of “geriatric dentistry” (Odontogeriatria). This enhances the academic identity and postgraduate education, although it may pose challenges regarding equity (28).

In a country like India, where the population is large and aging rapidly, wider system integration could potentially provide broader health benefits to the population rather than relying solely on specialization.

### 5.3 Policy box 3: ethical governance in geriatric oral health education

Ethics are not merely supplementary to geriatric oral health education; they are fundamental. This is because a significant portion of older adults experience cognitive impairment, sensory alterations, or dependence, which further complicates conventional notions of consent and autonomy. Consequently, these curricula should equip students with the skills to navigate ethical complexities while ensuring the dignity of patients is preserved (1).

**Core governance principles encompass:**

- Supported decision-making: Teaching students how to assess decision-making capacity and involve family caregivers or legal representatives in a manner that aligns with ethical and legal principles (1).
- Proportionality of care: Utilizing the Toco guide to guide treatment planning, which balances clinical benefits against burdens, prognoses, and patient values (16).
- Patient safety and supervision: Establishing clear boundaries for student supervision and determining the extent of autonomy they can exercise in geriatric clinics to minimize risks (31).

Simulation-based education offers a policy-relevant approach to these ethical challenges (20). It allows learners to rehearse complex scenarios in a safe, controlled environment before interacting with vulnerable patients.

### 5.4 Policy box 4: practice implications for clinicians and health systems

Policy and educational reforms ultimately translate into practical consequences that impact clinicians, patients, and health systems (11).

For clinicians, enhanced geriatric and interprofessional education broadens their professional roles beyond routine care to encompass preventive counseling, functional assessment, and care coordination. Dentists actively contribute to geriatric teams, enhancing continuity and the quality of care (32).

Patients, along with their caregivers, benefit from a more integrated care strategy that addresses oral health. This approach reduces unmet needs, improves comfort and nutrition, and ultimately enhances overall quality of life. Teamwork practices are crucial in facilitating communication and shared decision-making, especially for the cognitive-impaired population (8).

Therefore, improved oral health among the elderly can lead to reduced avoidable hospitalizations (37), postponed long-term care needs, and alignment with the objectives of value-based care (10, 11). This strategy aligns well with the goals of the health system.

### 5.5 Practice implications box: translating policy into action

- Integrate oral health assessments into routine geriatric and primary care workflows (23).
- Mandate interprofessional clinical exposure in geriatric settings during dental training (31).
- Align accreditation standards with national aging policies to reinforce educational priorities (17).
- Invest in faculty development to maintain interprofessional and geriatric curricula (6).
- Leverage digital health and teledentistry to enhance access to underserved older populations (13).

These policy boxes collectively emphasize that geriatric oral health education is a crucial link between policy intentions and clinical realities. Without explicit governance, ethical safeguards, and system integration, educational reform is unlikely to achieve its intended impact (29, 33).

## 6 Actionable recommendations

Actionable recommendations are the cornerstone of every Policy & Practice Review. These recommendations are the results of the analysis, presented as concrete steps that can be taken. They should not only be well-informed, like the ideas discussed in the analysis, but also feasible within the existing governance framework and the constraints of the healthcare system. In light of the policy analysis and its implications outlined above, the following multi-level policy recommendations have been developed. These recommendations are intended to be applicable within the Indian setting, although they are generally globally applicable in aging societies.

### 6.1 Recommendations for accreditation and regulatory bodies

Accreditation and regulation are among the most potent tools for driving enduring change in education. Meeting accreditation requirements leads to institutionalization in curricula, testing, faculty, and resource allocation. Conversely, voluntary measures often become less noticeable.

#### 6.1.1 Establish explicit competency statements in geriatric oral health

A central debate in geriatric reform is whether to prioritize high-level specialization or broad-based undergraduate competence. From a health system perspective, academic reform at the undergraduate level must be the initial priority.

While specialized fellowships are necessary for clinical leadership, a “specialist-only” model is unsustainable for a country with India's demographic volume. Relying solely on a small cadre of specialists creates a bottleneck, leaving the vast majority of community-dwelling and institutionalized seniors without basic care (38). Therefore, the first strategic step is the institutionalization of geriatric oral health as a core competency for all dental graduates.

Currently, undergraduate dental students often do not receive an adequate level of readiness for comprehensive geriatric care. Dental education is often procedure-based, with insufficient emphasis on the systemic and cognitive changes that occur in the aging population. For instance, undergraduate students often demonstrate gaps in the following areas:

- Systemic Co-management: Managing the effects of multimorbidity, such as the effects of polypharmacy on the oral cavity (37).
- Cognitive Decline: Managing ethical consent issues and behavioral management in dementia patients.
- Collaborative Practice: Working with medical colleagues to ensure patient safety in an interprofessional context (33).

It is advisable that a specific set of geriatric oral health competencies in the accreditation standards for undergraduate and postgraduate dental courses. These competencies should encompass not only age but also functional age, frailty, cognitive functions, nutrition, and polypharmacy (16). To ensure accountability, measurable attributes with direct links to professional activities have been emphasized (15). Unless there is a move away from classroom-based education to clinical IPE, the undergraduate student will not be adequately prepared to consider the patient's oral cavity in the context of the whole patient. This is the starting point for a level of safety and equity, upon which further specialization can be developed (32). Furthermore, there is a recommendation for Australian University Dental Schools to adopt these changes and implement them through models for education that are being developed at various universities for different groups of stakeholders (14). Additionally, it is suggested that curriculum renewal should be considered by including skills for effective communication.

#### 6.1.2 Mandate interprofessional clinical education in geriatric environments

A standard of accreditation could encompass supervised clinical exposure in a real-world geriatric clinical setting, such as a long-term care facility, geriatric outpatient clinic, or elder care program. While simple classroom interprofessional education is not sufficient to foster collaborative competency, clinical interprofessional education has been linked to a deeper understanding of the roles of the personnel involved and improved patient safety outcomes (29, 31).

#### 6.1.3 Align licensing and exit exams with the geriatrics complexity

Licensure examinations should incorporate case-based assessments that reflect real-world geriatric scenarios, such as multimorbidity, medication interactions, cognitive impairment, and ethical consent (6, 18).

This assessment reform communicates to institutions and learners that geriatric competence is a fundamental professional expectation, not a secondary concern.

### 6.2 National policy and governance recommendations (India-focused)

To scale educational change, national policy integration is crucial. Incorporating oral health into aging and primary care policies is essential for creating favorable conditions for dental education. Additionally, ensuring topic relevance is vital.

In the Indian scenario, the major challenge is the historical divide between dental education and the overall public health paradigm. To overcome this gap, the following recommendations can be made.

#### 6.2.1 Include oral health in the national programme for health care of the elderly (NPHCE)

NPHCE guidelines should be revised to address screening, counseling, and referral practices related to dental health. By linking dental colleges and teaching institutions directly with geriatric districts, we can facilitate student rotations and enhance geriatric dental services (21, 22). This approach will promote interprofessional education and better meet the population's dental needs.

**Curricular mandates:** The Dental Council of India (DCI) must go beyond organizing workshops and incorporate geriatric competencies as a mandatory and examinable part of the undergraduate Bachelor of Dental Surgery (BDS) curriculum. This is necessary to align with the National Programme for Health Care of the Elderly (NPHCE).

#### 6.2.2 Integrate geriatric oral health into national workforce planning and define the role of dentists in primary care for older adults

**Primary care integration:** Oral health screening needs to be made a part of the service delivery package of Ayushman Bharat Health and Wellness Centres. This requires training Mid-level Health Care Providers (MLHPs) in the use of basic geriatric assessment tools to identify patients at high risk for referral (23). The Ministry of Health and Family Welfare must clarify the role of dentists in multidisciplinary teams for elderly patients across primary, secondary, and tertiary health institutions. The dental profession's role in health and wellness centers under the Ayushman Bharat Scheme should acknowledge the inclusion of dental practitioners in treating the elderly in primary health institutions (23, 24).

#### 6.2.3 Focus on faculty development through national funding mechanisms

Sustainable curricular reform hinges on faculty capacity. National funding mechanisms should support faculty development in geriatric dentistry and interprofessional education through fellowships, certification programs, and structured continuing professional development. This investment can enhance faculty capacity without the immediate establishment of a separate specialty, which may not be feasible or equitable (6, 18).

##### 6.2.3.1 Recommendations for LMICs: scalability and task-sharing

While the recommendations above are specific to the Indian regulatory framework, the overall strategy of workforce optimization can be scaled up to other Low- and Middle-Income Countries (LMICs) with similar demographic shifts.

**Task-Sharing Models:** In areas with a shortage of dental specialists, educational initiatives should focus on training non-dental personnel (nurses, community health workers) to provide basic oral care and to recognize “red flags” indicative of dysphagia or xerostomia in vulnerable elderly populations (21).

**Low-Resource Interprofessional Education (IPE):** The application of IPE in the classroom, such as joint seminars for medical and dental students on the systemic effects of polypharmacy, provides a cost-effective starting point for developing a culture of collaboration without requiring immediate changes to clinical infrastructure (20).

##### 6.2.3.2 Globally transferable recommendations: systemic resilience

In addition to the particular needs of developing healthcare systems, there are a number of educational needs that are universally applicable and necessary for the development of a resilient, age-ready healthcare workforce.

**Clinical IPE in Long-Term Care:** The “gold standard” of interprofessional education is to require formalized interprofessional rotations in geriatric departments or nursing homes, which has been proven to decrease complications such as aspiration pneumonia and to decrease the total cost of long-term care by maintaining nutritional and systemic health (10, 26, 39, 40).

**Competency-Based Standards:** Accreditation bodies worldwide should adopt the IPEC Core Competencies (2016) update to ensure that oral health professionals are prepared for “Value-Based Care” in aging populations, emphasizing roles and responsibilities within a multi-disciplinary team (32).

### 6.3 Recommendations for academic institutions and universities

Academic institutions are the places where policies are put into action. The way leadership is structured, how governance operates, and how faculty members interact with students all contribute to the institution's success.

#### 6.3.1 Longitudinal, community-engaged geriatric rotations

Dental schools should not limit their teaching to a single, comprehensive look at older patients. Instead, they should offer long-term rotations that allow students to observe real people in various care settings over time. This continuity will enable students to comprehend the progression of chronic diseases, changes in functional abilities, and the role of caregivers (6, 22).

#### 6.3.2 Ensure interprofessional education is a sustained curricular framework

Interprofessional education (IPE) should not be confined to isolated modules; it should seamlessly integrate throughout the entire curriculum. Incorporate shared case discussions, joint simulations, and collaborative clinical experiences in a manner that fosters competence over time and helps students develop their professional identities (31).

#### 6.3.3 Utilize simulation to develop ethical and safety competencies

Simulation-based learning should be systematically introduced to train students for the ethical complexities that arise in geriatric scenarios, such as supported decision-making, end-of-life care, and communication with cognitively impaired patients and their families (20). This approach would enhance patient safety without compromising educational rigor.

#### 6.3.4 Institutional accreditation

The National Assessment and Accreditation Council (NAAC) and NABH must include interprofessional geriatric clinical rotations as a specific quality parameter for teaching hospitals and dental institutions.

### 6.4 Health systems and financing to support prevention

Strengthened education reform involves building the necessary health system structures and funding models that reward prevention, continuity, and teamwork.

#### 6.4.1 Reworking reimbursement to support preventive and collaborative geriatric dental care

Public and private insurers should cover preventive oral health assessments, management of oral function, and team-based care within geriatric pathways. International experience shows that this payment model encourages collaboration and leads to better outcomes (25, 26).

#### 6.4.2 Increasing access to integrated dental services within long-term care

Health systems must develop contracts and organizational structures that enable dentists to join interdisciplinary teams serving long-term care facilities. These arrangements reinforce skills acquired through interprofessional education and enhance the continuity of care for vulnerable older adults (22, 27).

##### 6.4.2.1 The role of mobile dental services in geriatric care

A major barrier to effective geriatric oral health care is the so-called “mobility gap.” It is characterized by declining physical capabilities, cognitive impairments, and transportation difficulties that prevent older adults from accessing conventional oral health care services (38). Mobile Dental Services is considered an effective policy intervention that facilitates the transition from emergency response to proactive prevention. By performing bedside screenings and offering professional oral hygiene care at long-term care facilities or private residences, Mobile Dental Services reduces bacterial loads that contribute to systemic complications. High-level evidence supports that Mobile Dental Services is the first line of defense against aspiration pneumonia, which is a major preventable cause of geriatric hospitalizations and deaths (10).

Apart from health outcomes, Mobile Dental Services contributes to the sustainability of health systems by filling the service gap between urban and rural areas (39). From an economic point of view, community-based oral health care through domiciliary care maintains physical function, prevents frailty, and in super-aged countries, long-term care costs are reduced as malnutrition and systemic infections are prevented, which are common reasons for institutionalization (26).

Mobile Dental Services provides an opportunity for Interprofessional Education (IPE) and interprofessional practice. Mobile Dental Services care requires an interprofessional approach, where dentists, nurses, pharmacists, and social workers work together to manage polypharmacy, cognitive decline, and other conditions. Mobile Dental Services care in India can be linked to the National Programme for Health Care of the Elderly (NPHCE) for decentralization of specialist care to the community, as envisaged by Ayushman Bharat, for social accountability of dental institutions (23, 24).

### 6.5 Globally applicable strategies and readiness for the future

While national contexts differ, several strategies are broadly applicable across health systems.

#### 6.5.1 Employing age-friendly frameworks like the 4Ms

The 4Ms framework—What Matters, Medication, Mentation, and Mobility— provides a proven, age-appropriate structure for incorporating oral health into geriatric education and clinical practice. *What Matters* ensures that oral healthcare aligns with older adults' values, preferences, and quality-of-life goals, promoting a person-centered approach to treatment planning. *Medication* addresses the high prevalence of polypharmacy in older adults and its oral health repercussions, such as xerostomia, increased caries risk, and other medication-related oral complications. This underscores the significance of interprofessional collaboration. *Mentation* focuses on cognitive and mental health conditions that impact oral hygiene practices, communication, consent, and adherence to care, necessitating adapted clinical strategies and caregiver involvement. *Mobility* highlights physical limitations that may hinder access to dental services and affect daily oral hygiene and masticatory function (31).

Overall, the 4Ms framework serves as a common language across professions and care settings, supporting the harmonization of education, practice, and policy. It facilitates the integration of oral health into comprehensive, age-friendly healthcare systems.

#### 6.5.2 Invest in evaluation and outcomes research

Policy makers and educators should support long-term evaluation of the geriatric and interprofessional curricula, including tracking graduate practices and patient outcomes. Robust evaluation strengthens the evidence base and informs iterative policy refinement (32).

#### 6.5.3 Adopt digital health and teledentistry

Digital tools and teledentistry can expand interprofessional consultation and education to underserved and rural communities. Building digital competencies into geriatric training prepares graduates for new care models and supports health equity (13).

## 7 Discussion: geriatric oral health education: a critical juncture

Geriatric oral health education currently exists at a critical juncture related to global demographic change, healthcare reform, and accountability in healthcare professionals. This comes at a point in time when elderly patients are increasingly presenting with highly complex medical, functional, and social care needs, making traditional models of healthcare professional education, often concentrated on procedure and interprofessional divides, ill-suited for safe, equitable, and holistic provision. The synthesized results indicate that improving geriatric oral health educational reform is much more than an educational challenge—it has broad strategic implications in terms of its very impact on successful public health and healthcare system viability (37).

### 7.1 Designating geriatric oral health education resources within the health infrastructural framework

One of the most crucial points highlighted in this review is the significance of incorporating geriatric oral health education into curricula. It is not merely an enhancement of existing programs but a fundamental aspect of the functioning of healthcare systems. Various factors, such as aging, dietary needs, chronic diseases, vulnerable populations, and quality of life, intersect when it comes to oral health in older individuals.

Educating healthcare professionals directly impacts their performance. Healthcare systems that neglect these specific geriatric skills are bound to face consequences, including unnecessary hospital admissions, vulnerable seniors aspirating fluids, a higher susceptibility to pneumonia, and an earlier onset of functional impairment (9, 10). This underscores the detrimental impact of neglecting geriatric education on healthcare efficiency. From a policy perspective, Education serves as a preventive strategy with long-term benefits.

### 7.2 Interprofessional education as a policy instrument, not an educational add-on

With the development of an Interprofessional Education module, it is evident that the primary level of care is the most feasible and impactful level for initial implementation. It is at this level that it is possible to move from awareness of interprofessional education to actual implementation of interprofessional practice, especially in community and primary health center settings, such as the Ayushman Bharat Health and Wellness Centres found throughout India, which are designed to provide preventive screening, early intervention, and management of chronic multimorbidity (20). Implementation of IPE at this level allows dental students to work with mid-level healthcare providers and primary care physicians to address issues of oral-systemic relationships of aging, such as those seen with diabetes and periodontal disease, before these issues become tertiary-level complications. Not only will it improve sustainability of the health system by reducing preventable hospitalizations for conditions such as aspiration pneumonia (10), but it will also support global mandates for universal health coverage by integrating specialized oral health expertise with existing primary care providers (12, 38).

Authors reiterate the importance of viewing inter-professional education (IPE) as a policy agenda rather than an educational choice. There is substantial evidence suggesting that IPE fosters collaboration and improves patient outcomes when implemented over the long term and based on experience. However, without a robust policy and financing structure, IPE activities are prone to fragmentation and sustainability challenges (20, 29, 31). Since geriatric practice inherently involves teamwork, the absence of organized IPE exacerbates confusion within each role and hinders effective communication. By integrating IPE within accreditation and licensing criteria, collaboration and teamwork can become professional requirements. This aligns with the World Health Organization's recommendations for healthcare workforce development during transitions in the aging population (1, 29).

### 7.3 India's policy opportunity: from demographic risk to reform catalyst

India stands at a crossroads. A rapidly ageing population with a rise in multimorbidity, combined with high out-of-pocket health expenditures, presents a risk and an opportunity. To continue to marginalize oral health within national ageing policies represents the loss of an opportunity to use dental education as a means of strengthening the health system (21, 23).

However, the country's centralized regulatory framework also presents a unique advantage. Small, targeted modifications to national accreditation rules and policy guidance can swiftly impact hundreds of institutions, enabling a scalable transformation of curricula. By integrating geriatric oral health competencies into established programs such as the National Programme for Health Care of the Elderly and Ayushman Bharat, reforms can be implemented without the creation of parallel systems. As observed in Japan and Brazil, embedding these competencies within a broader care framework tends to yield fairer and more equitable outcomes compared to focusing solely on specialty care (25, 28).

The National Medical Commission (NMC), formerly known as the Medical Council, plays a vital role in moving inter-professional initiatives forward from pilot stages to full-scale regulation through the mandatory requirement of geriatric oral health as a core competency for medical undergraduates. The NMC can ensure that future medical graduates are able to recognize dental “red flags” during routine geriatric patient screenings through the mandatory requirement of oral health screenings, which include the assessment of xerostomia and periodontic-systemic relationships within the Competency-Based Medical Education (CBME) model. The NMC can work in tandem with the Dental Council of India (DCI) in co-regulating joint medical and dental clinical rotations in order to ensure that medical interns and dental students receive academic credit for inter-professional practice in settings of high patient need, such as geriatric patient care facilities and community health centers. This co-regulatory relationship is vital in order to break down professional barriers and foster an inter-professional attitude that oral health is not a peripheral specialty in itself but an essential component of patient care and nutrition in the elderly patient population (37, 33).

### 7.4 Equity, access, and ethical dimensions

Equity is the cornerstone of geriatric oral health policy. Economically disadvantaged, immobile, or cognitively challenged older adults bear a heavier burden of oral disease and face significant barriers to accessing care. Community-based models of education often prioritize home-based care and integration with long-term care facilities, effectively addressing these gaps (22, 36).

Ethically, training students to provide services to vulnerable seniors necessitates explicit attention to consent, autonomy, and appropriate levels of care. Simulation and supervised clinical exposure are essential in developing these skills while safeguarding patient welfare (20). From a governance standpoint, ethical preparedness should be regarded as a core accreditation outcome, rather than an implicit expectation (16).

### 7.5 Implementation challenges and system constraints

While the proposed policy and education reform are evidence-based, their actual implementation faces practical challenges. Faculty shortages, overcrowded curricula, and a shortage of clinical training sites are commonly cited obstacles. Mandates without dedicated funding and continuous faculty development risk evolving into superficial compliance without substantial, sustained change (6, 18). System-level constraints, such as fragmented financing and workforce gaps, further complicate the integration of these reforms. Alignment of reimbursement models with preventive and collaborative geriatric oral health services is crucial to sustain both educational reform and practice transformation (11). Policymakers must recognize that a mismatch between education and service delivery undermines outcomes in both areas (22).

### 7.6 Implications for research and evaluation

This policy review not only highlights areas that require refinement based on existing knowledge but also identifies unexplored areas that warrant further investigation. Longitudinal research is crucial to understand the long-term impacts of geriatric or interprofessional education on practice, outcomes, and costs for a generation of graduates. This information would validate political agendas and help establish standards. A mixed methods approach that captures experiences in learning, teaching, and as a patient could shed light on the local factors that either support or hinder implementation. In developing countries, implementation studies are particularly important to adapt global approaches to local contexts (18).

### 7.7 Moving towards policy coherence on education, health, and social care

To achieve more meaningful and enduring changes, it appears that there's a need for convergence among the education, healthcare, and community sectors. Enhanced oral education for the elderly seems to be at the very intersection of these streams and could serve as an example of an integrated workforce strategy (12, 17).

This policy review highlights the necessity to move away from the “silo mentality” of each discipline and adopt a future-oriented “life course” approach to strategies in the field of healthy aging. This approach, based on the coherence mentioned earlier, is crucial for addressing the complex needs of older individuals and achieving the healthy aging objectives outlined in various global documents.

## 8 Conclusion

Geriatric oral health education is at a critical juncture with respect to aging policy, health system effectiveness, and professional accountability. This review has reinforced that specialized education is not an optional curriculum addition but, rather, an essential core of an effective health system. The following conclusions are specifically designed to align with the study's three core objectives:

Firstly, it is evident that there is a prevailing “geriatric gap” where aging is recognized as a public health imperative, but oral health is still ignored as part of broader long-term care and family policy development. In India, despite having an existing infrastructure of dental colleges, it is evident that there is a prevailing mismatch with respect to national initiatives such as the National Programme for Health Care of the Elderly (NPHCE). To ensure policy integration, it is imperative that health regulators shift beyond elective workshops and ensure that geriatric education is incorporated into mandatory accreditation criteria of the Dental Council of India (DCI) and National Assessment and Accreditation Council (NAAC), thereby transforming dental colleges into socially accountable players with respect to the Ayushman Bharat initiative.

Secondly, modern dental education is still procedure-oriented and fails to adequately prepare the workforce for the demands of multimorbidity and cognitive decline. The consequences of such a failing model are quantifiable and manifest in the increased incidence of aspiration pneumonia and long-term care costs. To redress this failing model requires a learning approach that prioritizes community engagement and ethical practice, in order to ensure the most disadvantaged older adults, who are functionally dependent, receive equitable care.

Thirdly, Interprofessional Education (IPE) is the primary mechanism through which a health system is strengthened. As witnessed in the Japanese and Brazilian models, the most effective form of IPE is one which progresses from a model of “classroom-based exposure” towards an integrated model of practice. By learning “with, from, and about” other health professionals, dental students are able to acquire the skills in communication and role definition which are required for cohesive working in the geriatric wards and home-care environments. Such a model is the key to improving patient safety and attaining the triple aim of health care.

In summary, the Indian scenario provides both a warning and a blueprint. The pace of demographic aging necessitates an urgent shift toward integrated models of care. By weaving geriatric and interprofessional competencies into the fabric of dental education, policymakers and educators can ensure that the dental profession remains relevant and capable of supporting healthy aging at scale.
